# Response to: “Emergency Medicine Physician Assistant Postgraduate Training Programs: Program Characteristics and Training Curricula”

**DOI:** 10.5811/westjem.2019.12.43459

**Published:** 2019-04-23

**Authors:** Chadd Kraus, Terry E. Carlisle, Devin M. Carney

**Affiliations:** * Geisinger Health System, Department of Emergency Medicine, Danville, Pennsylvania; † University of Missouri-Columbia, Department of Emergency Medicine, Columbia, Missouri; ‡ University of Missouri-Columbia, Columbia, Missouri

Dear Editor:

We thank Mr. Wu, PA-C, MHS (President, the Society of Emergency Medicine Physician Assistants [SEMPA]) for his insightful letter in response to our paper, “Emergency Medicine Physician Assistant (EMPA) Postgraduate Training Programs: Program Characteristics and Curricula.”^1^,^2^

As Mr. Wu notes, SEMPA continues to lead the EM specialty-specific training of EMPAs, including through their postgraduate training and practice standards.^3^,^4^ The discussion in our paper was approached from the perspective and framing of EMPA training and certification in the context of physician postgraduate training found in EM residency programs. As our results suggest, there are some similarities, although variability remains in EMPA training program curricula. The rapid growth of EMPA postgraduate programs highlights the appetite for this training and underscores the need to establish formalized educational and curriculum standards such as those developed by SEMPA to provide structure and quality assurance for new and old programs alike. As with EM residency and fellowship programs for physicians, accreditation and certification are key to ensuring the best education and training for EMPAs in the context of a rapid expansion in the number of EMPA programs.

The training standards developed by EMPA program directors and endorsed by SEMPA are voluntary. The next step in the evolution of EMPA postgraduate training would be for these SEMPA standards to become codified in a way that EMPA training programs would be required to use, in the same way that accredited EM training programs use the “Model of the Clinical Practice of Emergency Medicine,”^5^ which outlines the core content of knowledge, skills, and abilities expected of an emergency physician certified by the American Board of Emergency Medicine.

The intent of our paper was to compare the current pathway for EMPA postgraduate training to EM residency training. While EMPAs have continuing education requirements related to their national certification and state licensure and can earn and maintain certificate of added qualification in EM, there remains an opportunity for the development of formal certification and maintenance of certification (analogous to board certification for emergency physicians) for those PAs who have completed EM-specific postgraduate training or who might be “grandfathered” via a practice track. While the accreditation process of the Accreditation Review Commission on Education for the Physician Assistant (ARC-PA) is in abeyance, our opinion is that an accrediting organization similar to the Accreditation Council for Graduate Medical Education for medical residencies could accomplish the goal of required, rather than voluntary, standards for EMPA training.

We agree with SEMPA that there remain several pathways to becoming an EMPA, including practice-based training. However, in the future, workforce and market demands might require formal training in order to be competitive from an employment standpoint, although that is currently not the case. Additionally, formal training might allow EMPAs to recognize their unique and specialized training and skills and to distinguish themselves from other providers in EM, and from PAs in other fields of medical practice.

We agree with Mr. Wu and SEMPA that EMPAs are invaluable members of the EM workforce now and into the future. And we applaud the ongoing work of SEMPA in the development of EMPA training. We hope that our research provides a foundation for future development of standardized, accredited training programs for emergency medicine physician assistants.

## References

[1] Kraus CK, Carlisle TE, Carney DM (2018). Emergency Medicine Physician Assistant (EMPA) postgraduate training programs: program characteristics and training curricula. West J Emerg Med.

[2] Wu F (2019). Letter to the Editor: Emergency Medicine Physician Assistant (EMPA) postgraduate training programs: program characteristics and training curricula. West J Emerg Med.

[3] Society of Emergency Medicine Physician Assistants (SEMPA) (2015). Emergency Medicine Physician Assistant Postgraduate Training Program Standards.

[4] Society of Emergency Medicine Physician Assistants (SEMPA) Emergency Medicine Physician Assistant Postgraduate Training and Emergency Medicine Physician Assistant Practice Guidelines.

[5] The American Board of Emergency Medicine (ABEM) 2016 Model of the Clinical Practice of Emergency Medicine.

